# Genetic structure and symbiotic profile of worldwide natural populations of the Mediterranean fruit fly, *Ceratitis capitata*

**DOI:** 10.1186/s12863-020-00946-z

**Published:** 2020-12-18

**Authors:** Katerina Nikolouli, Antonios A. Augustinos, Panagiota Stathopoulou, Elias Asimakis, Anastasios Mintzas, Kostas Bourtzis, George Tsiamis

**Affiliations:** 1 Insect Pest Control Laboratory, Joint FAO/IAEA Programme of Nuclear Techniques in Food and Agriculture, A-1400 Vienna, Austria; 2 Department of Biology, University of Patras, 26504 Patras, Greece; 3 Present address: Department of Plant Protection, Hellenic Agricultural Organization-Demeter, Institute of Industrial and Forage Crops, 26442 Patras, Greece; 4 Department of Environmental Engineering, University of Patras, 30100 Agrinio, Greece

**Keywords:** Medfly, Microbiota, Bacterial communities, Microsatellites, 16S rRNA, Illumina sequencing

## Abstract

**Background:**

The Mediterranean fruit fly, *Ceratitis capitata,* is a cosmopolitan agricultural pest of worldwide economic importance and a model for the development of the Sterile Insect Technique (SIT) for fruit flies of the Tephritidae family (Diptera). SIT relies on the effective mating of laboratory-reared strains and natural populations, and therefore requires an efficient mass-rearing system that will allow for the production of high-quality males. Adaptation of wild flies to an artificial laboratory environment can be accompanied by negative effects on several life history traits through changes in their genetic diversity and symbiotic communities. Such changes may lead to reduced biological quality and mating competitiveness in respect to the wild populations. Profiling wild populations can help understand, and maybe reverse, deleterious effects accompanying laboratory domestication thus providing insects that can efficiently and effectively support SIT application.

**Results:**

In the present study, we analyzed both the genetic structure and gut symbiotic communities of natural medfly populations of worldwide distribution, including Europe, Africa, Australia, and the Americas. The genetic structure of 408 individuals from 15 distinct populations was analyzed with a set of commonly used microsatellite markers. The symbiotic communities of a subset of 265 individuals from 11 populations were analyzed using the 16S rRNA gene-based amplicon sequencing of single individuals (adults). Genetic differentiation was detected among geographically distant populations while adults originated from neighboring areas were genetically closer. Alpha and beta diversity of bacterial communities pointed to an overall reduced symbiotic diversity and the influence of the geographic location on the bacterial profile.

**Conclusions:**

Our analysis revealed differences both in the genetic profile and the structure of gut symbiotic communities of medfly natural populations. The genetic analysis expanded our knowledge to populations not analyzed before and our results were in accordance with the existing scenarios regarding this species expansion and colonization pathways. At the same time, the bacterial communities from different natural medfly populations have been characterized, thus broadening our knowledge on the microbiota of the species across its range. Genetic and symbiotic differences between natural and laboratory populations must be considered when designing AW-IPM approaches with a SIT component, since they may impact mating compatibility and mating competitiveness of the laboratory-reared males. In parallel, enrichment from wild populations and/or symbiotic supplementation could increase rearing productivity, biological quality, and mating competitiveness of SIT-important laboratory strains.

**Supplementary Information:**

The online version contains supplementary material available at 10.1186/s12863-020-00946-z.

## Background

The Mediterranean fruit fly, *Ceratitis capitata* (Diptera: Tephritidae) is a cosmopolitan species that affects numerous fruit crops worldwide [1]. Females oviposit their eggs in the mesocarp of the fruit and this results in economic loss because, following hatching, larvae feed on the fruit reducing both yield and value of the product and, in addition, oviposition holes facilitate secondary fungal and bacterial infections [2]. Due to the heavy economic losses if left untreated, several approaches are being followed to reduce the economic impact of this agricultural pest.

In the last decades, emphasis has been put on control strategies that have the least possible negative impact on environment. Among them, the sterile insect technique (SIT), as part of the area-wide integrated pest management (AW-IPM) is considered as a species-specific, environmentally friendly control method [3]. SIT is in principle based on the release of sterile insects of the targeted species, ideally only males, to suppress or, even better, eradicate a targeted population. In this approach, the sterility is delivered through irradiation [4]. Therefore, after irradiation and release, sterile males are expected to mate with the females of the targeted population thus leading to infertile crosses and subsequent population reduction. Medfly has been the model for the design of control strategies that have an SIT component with the VIENNA 7 and VIENNA 8 genetic sexing strains (GSS) being used in all mass rearing facilities worldwide facilitating male-only releases [5, 6]. A primary challenge to be addressed before any SIT application is the maintenance of a high biological quality laboratory population that will retain adequate male mating competitiveness during SIT releases in the field, keeping at the same time mass rearing cost-efficient. Therefore, irrespectively of the strain(s) that will be used, the major objective is to have robust, fit, and competitive laboratory strains. These are not easy to retain under continuous artificial rearing and especially mass rearing conditions, which may be suboptimal in different ways.

The presence of genetically differentiated populations can lead to reduced mating compatibility between laboratory strains and SIT-targeted populations [3]. Genetic structure of medfly natural populations has been extensively studied. All studies suggest the African origin of the species and there are certain pathways documented regarding its ‘out of the Africa’ expansion, including both established populations and recent invasions [7–13]. The recent availability of an advanced genome assembly for the medfly [14], along with the gradual decrease in sequencing cost is expected to deliver genome-wide and quick approaches to more thoroughly study recent invasions and population structuring. Currently, there are so far no documented cases of unsuccessful SIT efforts (or of reduced efficiency) due to population structuring; however, the species has not been sampled across its range.

Being a SIT model for tephritids, medfly has been a target of extensive research in areas related to improvement of both rearing efficiency and biological quality of released males. Lots of resources have been invested in identifying the importance of gut microbiota [15]. Studies from different research laboratories have shown that certain bacterial strains can improve parameters important either for the productivity of the colonies (such as enhanced pupation rate, adult emergence, and fecundity) or for the biological quality of released males (such as flight ability, survival, and mating competitiveness) [16–24]. Only a few studies have focused on the structure of the symbiotic communities of domesticated and wild populations. Findings up to now indicate that a) Gammaproteobacteria and mainly different Enterobacteriaceae genera constitute the gut bacterial communities of the medfly, b) origin, developmental stage, and age are important for the gut bacterial profile structuring, c) the degree of domestication and rearing conditions are influential for the gut microbiota profile of laboratory populations, and, d) laboratory strains that are reared totally artificially harbor less diverse gut symbiotic communities than the wild populations [17, 19, 22, 25–28]. Despite advances, the gut bacterial communities of natural populations are rather poorly studied, and a rather limited range of bacterial isolates has been isolated and tested as candidate probiotics to support artificial rearing of medfly for SIT applications [16, 19, 22–24].

To ensure the efficacy of SIT applications, the biological quality and the continuous improvement of the mass-produced insects are of major importance. Adapting to the artificial environment poses considerable selection pressures on insects that may significantly alter both the genomic and the symbiotic profile. As a result, insects that will differ significantly from their wild counterparts, will be produced. Thus, it is required to develop a strategy that will allow maintaining the genetic and symbiotic diversity and preserve the ‘*wild’* character of the mass-reared colony. Refreshing the mass-reared strains periodically with wild material is a strategy that has been suggested to mitigate genetic issues occurring during mass-rearing [29–31]. Among other factors, the genetic and symbiotic profile of the wild material should be known beforehand, to avoid any phenomena of out-competition or quick performance decline observed in the past [30, 32]. Up to now, there are no studies in tephritids addressing both the genetic and the bacterial structure of natural populations. Such studies are important to understand whether the genetic and the bacterial profile are influencing each other and how. Understanding the interplay of these two factors will guide future decisions on the wild material that can be occasionally used to refresh the mass-reared colonies [29–31]. The present study aims to fill that gap by presenting the genetic structure and symbiotic profile of medfly populations collected from different countries worldwide.

## Results

### Analysis of the genetic structure of medfly natural collections

#### Polymorphism analysis

A total of 408 individuals belonging to fifteen different natural populations were analyzed (Additional file 1 Table S1), with a mean of 27.2 individuals per sample, ranging between 9 and 50. An average of 3.22 alleles (Na) per population was found, ranging between 1.85 and 4.25 (Table 1). A better estimation of population diversity is provided by the effective allele number (Ne), since both sample size, allele number, and relative representation in the population gene pool are considered and it was 2.02 per population. The average observed heterozygosity was 0.417, similar to the expected heterozygosity (0.410). Deviations from Hardy-Weinberg Equilibrium (HWE) were observed in almost all populations, in 33 out of the 120 population/marker combinations, ranging between zero and four markers per population (Table 1). Most of deviations were attributed to heterozygosity deficiency in different allele combinations which is consistent with either sub-structured populations or, most probably, with the presence of null alleles that can lead to misidentification of heterozygotes as homozygotes.

**Table 1:** Genetic diversity indices of 15 medfly collections

No		Population	Genetic analysis					
			N	Na	Ne	Ho	He	HWE
1	Europe	Greece1	30	2.87	1.61	0.429	0.325	2/8
2		Greece2	24	3.71	2.194	0.520	0.433	4/8
3		Spain	50	5.75	2.46	0.497	0.457	2/8
4		Croatia	29	4.25	2.09	0.404	0.388	4/8
5	Asia/Middle East	Israel	40	3.37	2.06	0.365	0.438	2/8
6	Australia	Australia1	20	2.25	1.66	0.293	0.339	4/8
7		Australia2	29	2.25	1.7	0.434	0.368	3/8
8	North America	Hawaii	24	3.71	2.43	0.563	0.517	1/8
9	Central America	El Salvador	24	3	2.05	0.367	0.434	2/8
10		Honduras	9	1.85	1.64	0.238	0.297	–
11		Nicaragua	29	3	2.01	0.453	0.452	1/8
12		Costa Rica	24	2.5	1.98	0.407	0.387	–
13	South America	Argentina	24	3.71	2.56	0.625	0.560	1/8
14		Brazil	28	3.87	2.33	0.548	0.476	4/8
15		Bolivia	24	2.25	1.45	0.110	0.284	3/8
*average*			*27.2*	*3,22*	*2.02*	*0.417*	*0.410*	

#### Microsatellite markers’ polymorphism

Markers presented different levels of variability, with 1.05 (*Ccmic14*) to 2.63 (*Ccmic32*) effective alleles per locus (Additional file 2 Table S2). Deviations from HWE were not evenly distributed to all loci, ranging between one (markers *Ccmic6, Medflymic30, Ccmic14*) and nine (*Ccmic32*) (Additional file 2 Table S2, Additional file 3 Table S3).

#### AMOVA

Molecular variance was analyzed to uncover the origin of the genetic variability observed. As evident from Additional file 4 Fig. S1, most of the variance (60%) is attributed to within individual differentiation, which is expected for highly polymorphic diploid markers, such as microsatellites. However, a substantial portion of the differentiation (29%) is attributed to differentiation among populations, clearly indicating the presence of differentiated populations within the dataset analyzed.

#### Genetic distances (Nei)

Genetic distances ranged between 0.032 (the two samples from Australia) and 1.105 (samples from Brazil and Honduras) (Additional file 5 Table S4). Geographic distance is important in the formation of genetic distances since the smaller values appear within certain geographic clusters, such as the Australian cluster and the Mediterranean cluster.

#### Population structuring

**Fig. 1:**
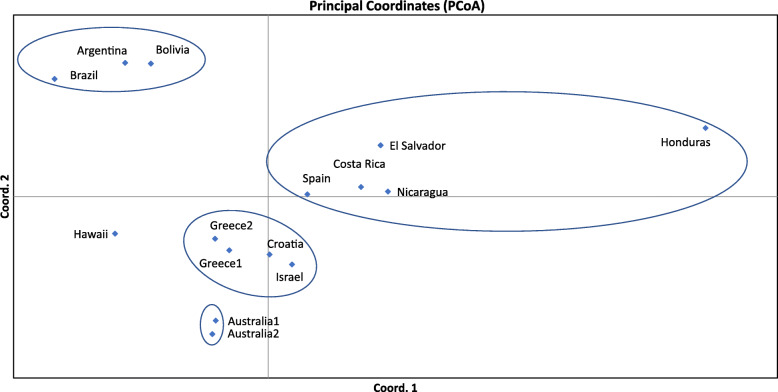
Principal Coordinates Analysis using Nei’s genetic distance matrix. Encircled populations are genetically more closely related

Nei’s genetic distance matrix was used to perform a PCoA analysis. As evident from Fig. 1, there is a clear genetic structuring of medfly populations, with the first three axis accounting for the 71% of the observed genetic differentiation. Samples from South America are clustered together (Brazil, Argentina, and Bolivia), quite apart from all other samples. The two samples from Australia seem to make a cluster of their own. Another group is formed by the eastern Mediterranean samples (Greece, Croatia, and Israel). Finally, the sole sample from Western Europe (Spain) is closer to the group formed by the Central American samples, while Hawaii is not clustered with any of the above groups.

**Fig. 2:**
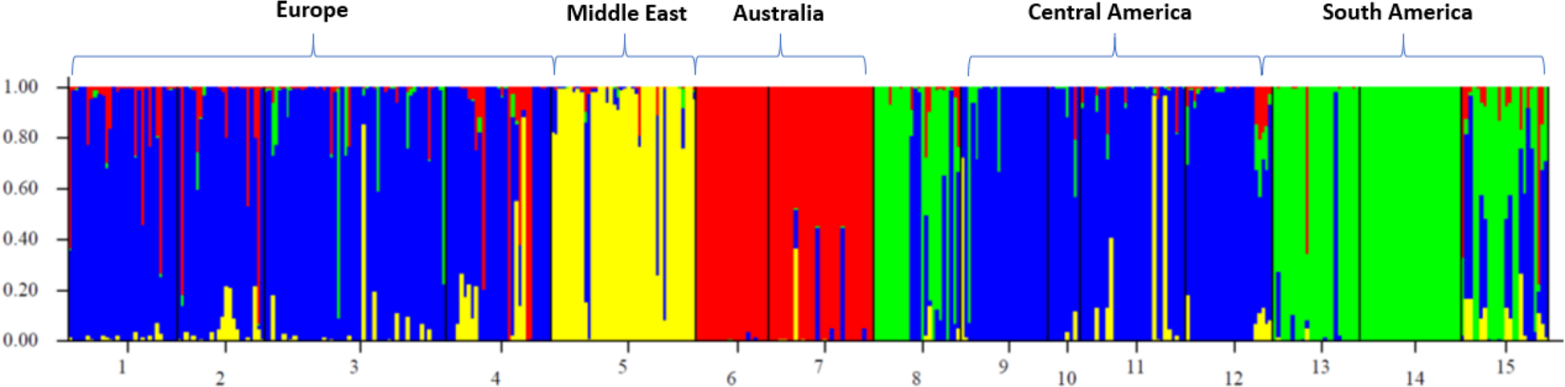
STRUCTURE clustering based on allele frequency variation, assuming the presence of four different groups. Individuals are represented by a vertical line and each color indicates a different cluster. *1: Greece1; 2: Greece2; 3: Spain; 4: Croatia; 5: Israel; 6: Australia1; 7: Australia2; 8: Hawaii; 9: El Salvador; 10: Honduras; 11: Nicaragua; 12: Costa Rica; 13: Argentina; 14: Brazil; 15: Bolivia*

A Bayesian analysis of the putative number of populations was performed with STRUCTURE software [33], following the modification suggested by Evanno and colleagues [34] (Additional file 6 Fig. S2). Our analysis points to the presence of at least four well-differentiated groups, quite similar to the PCoA clustering. As shown in Fig. 2, South America samples form one cluster (the ‘green’ cluster), the two samples from Australia cluster together (the ‘red’ cluster), while all Mediterranean and Central America samples form a 3rd cluster (the ‘blue’ cluster), with the exception of the sample from Israel that clusters apart from the other samples (the ‘yellow’ cluster).

### Analysis of bacterial communities

*16S rRNA gene* sequencing: A total of 8,318,552 16S rRNA gene sequences were generated from all samples. After filtering of low quality and chimeric sequences, 7,323,174 sequences were retrieved and used for downstream analysis. This equals to approximately 665,743 sequences per sampled population or 27,635 reads per sequenced individual. Analysis of alpha diversity and respective rarefaction curves indicate that sequencing depth was adequate to reveal the symbiotic diversity of the different samples (data not shown).

#### Overall symbiotic diversity of medfly natural populations

The overall microbiota diversity can be considered rather low. Three Phyla were the major components of the symbiotic communities of the different medfly samples. As expected, Proteobacteria were highly abundant, followed by small Firmicutes and minor Actinobacteria communities (relative abundances: 95, 4%, and less than 1% respectively) (Additional file 7 Fig. S3). At the Class level, only four classes were represented with relative abundances higher than 1%. More than 90% of the sequences belonged to Gammaproteobacteria and approximately 4% to Alphaproteobacteria. A small Bacilli community was present (~ 4%) and a minor Actinobacteria (~ 1%) (Additional file 7 Fig. S3B). Going down to genus level, only few genera were identified with relative abundances higher than 1%. Among them, different Enterobacteriaceae genera, known to dominate medfly bacterial communities were present, including *Klebsiella*, *Providencia*, *Tatumella*, *Citrobacter*, *Morganella*, *Enterobacter, Rahnella*, and *Pantoea* (in order of reduced relative abundance) (F Additional file 7 Fig. S3C). Interestingly, *Rahnella* sp. has been identified for first time in medfly natural populations with a relative abundance ranging from 0 to 8.3% (Additional file 7 Fig. S3C). Bacilli were represented mainly by a single genus (*Exiguobacterium*) and the same applied for Alphaproteobacteria (*Commensalibacter*) (Additional file 7 Fig. S3C).

#### Alpha diversity

Samples’ microbiota diversity was measured with species richness index and Shannon index. Number of observed OTUs ranged between 9 and 38 per sample (Additional file 8 Fig. S4A). Shannon index describes the variability in OTUs more accurately, since it takes into account both the number of OTUs and their relative abundance in the samples (Additional file 8 Fig. S4B). There are statistically significant differences in the bacterial diversity of the different species but there is not a pattern associated with specific factors, such as the geographic origin of the samples.

#### Taxa distribution

**Fig. 3:**
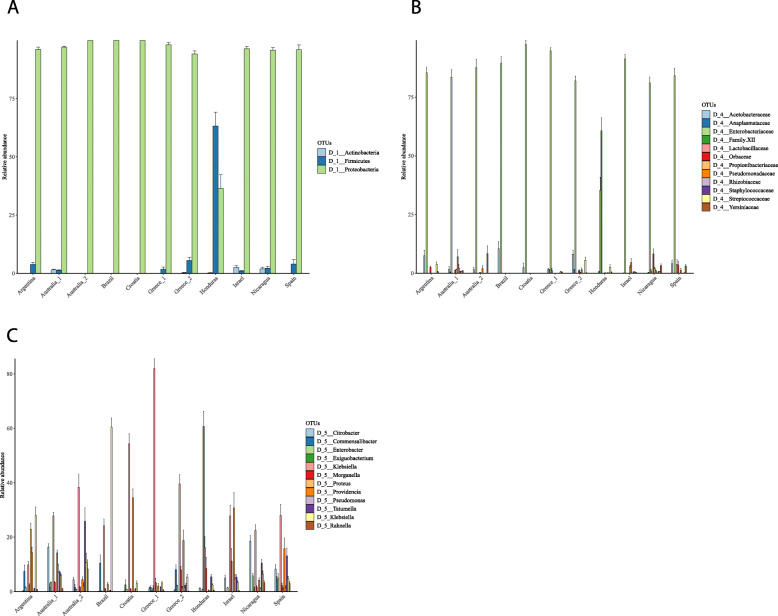
Diversity of the microbiota of the different medfly samples at **a** Phylum level, **b** Family level, and **c** Genus level

All samples were primarily dominated by Proteobacteria (more than 90% of the sequences in all samples), except for medflies from Honduras where Firmicutes had a higher relative abundance than Proteobacteria (63 and 36% respectively (Fig. 3a). Even at the family level, all samples share a quite similar microbiota profile, since the Enterobacteriaceae family accounted for more than 90% of the 16S rRNA gene sequences in all samples, while a single Firmicutes family was dominating Honduras’ sample (belonging to *Bacillales*), followed by Enterobacteriaceae (Fig. 3b). Despite the reduced overall diversity, differences among medfly samples become more evident at lower taxonomic levels. Going to genus level, there were no more than 12 different genera that substantially contribute to microbiota of all different samples. As expected from the higher taxonomic levels, Honduras’s sample was well differentiated since *Exiguobacterium* genus of Firmicutes showed the highest relative abundance (~ 60%), followed by different Enterobacteriaceae genera, such as *Klebsiella* (~ 18%) and *Morganella* (~ 5%). In most of the remaining samples, *Klebsiella* was the prevailing genus, followed by *Providencia* (Fig. 3c), except for Australia 2, Nicaragua, and Spain with *Rahnella* sp. being one of the most dominant taxa in the medfly populations from Australia 2 (Fig. 3c).

#### *Factors contributing to the structuring of medfly microbiota* profiles

**Fig. 4:**
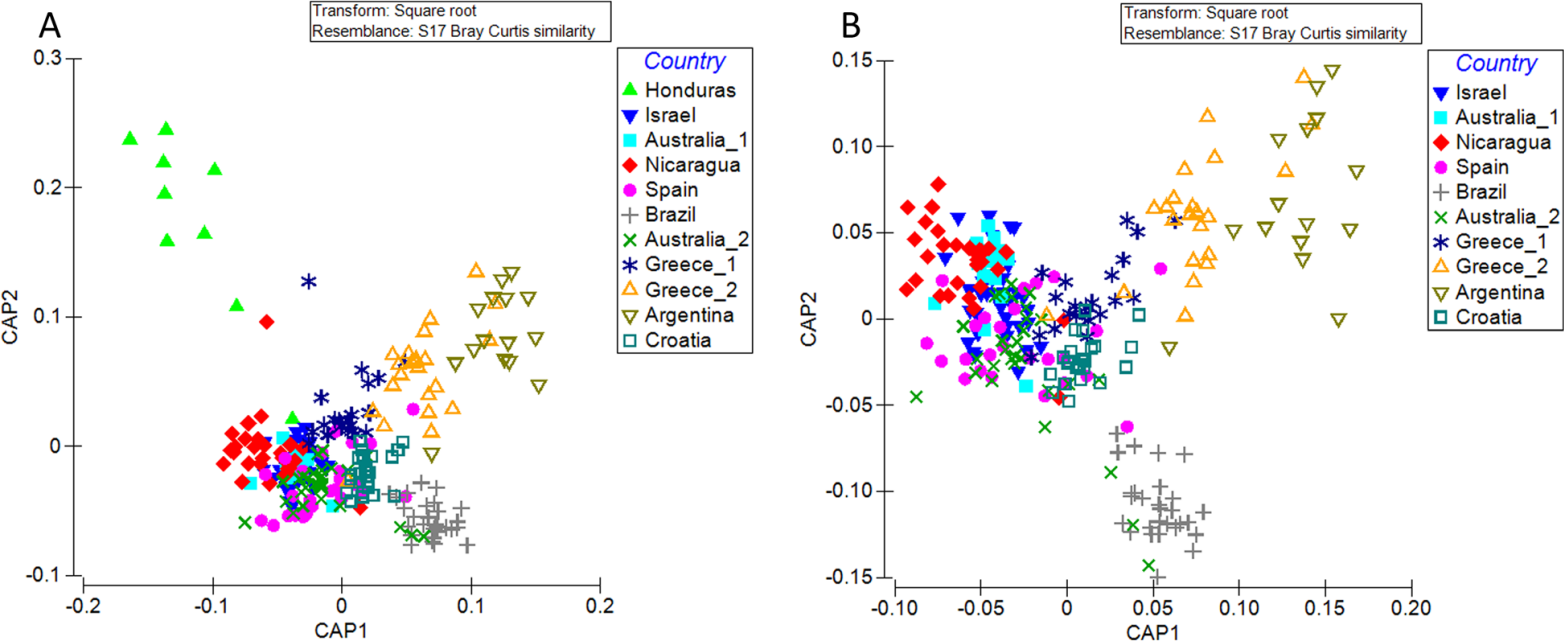
The CAP analysis of beta-diversity for **a** all 11 samples, **b** after excluding the sample from Honduras

As evident from the PERMANOVA analysis, pairwise comparisons indicated that the microbiota profiles of all samples are statistically different from each other (Additional file 9 Table S5), with medflies from Honduras being more differentiated from all other samples (Fig. 4). Therefore, both geographic origin and host seem to be important factors shaping microbiota profile of natural populations. However, the contribution of these factors cannot be really estimated under the setup of the present study. Asking whether sex is a significant factor in the structuring of the symbiotic communities, PERMANOVA analysis indicated that sex is not a significant predictor of the observed structuring (PERMANOVA; *p* = 0.111) (Additional file 10 Fig. S5).

#### Diversity beyond genus level

Even though few genera were dominant in our samples, analysis of OTUs beyond genus level showed that the same genera may be represented by different putative species in the different samples. Moreover, although most of them can coexist in the different populations, their abundances may vary a lot. This was evident in at least four of the most relatively abundant genera that are *Klebsiella, Providencia, Morganella,* and *Enterobacter* (Additional files 11, 12, 13, 14 Figs. S6–9).

## Discussion

### Main findings

Genetic analysis of fifteen medfly natural populations expanded our knowledge in previously poorly studied areas, such as those of Central and South America. Our results are in accordance with the main scenario of an African expansion of the species with the Mediterranean basin being the source of most of the subsequent invasions. At the same time, analysis of the symbiotic profile of eleven natural collections substantially contributed to our understanding of the structuring of bacterial communities since very limited information was available before (only populations from Israel and Italy have been analyzed so far). This analysis pointed to symbiotic communities of reduced diversity, with differential relative abundance and not differential OTUs presence, being the driving force of the bacterial profile structuring.

### Genetic structuring of medfly

As being a global pest, the invasion route and pathways of medfly have been well- studied in recent years [12, 13, 35, 36]. The expansion from Africa to Europe through Spain resulted in populations of reduced variability in the area [37]. The more recent expansion in Latin America and the Pacific seems to be consistent with independent and repeated invasions from both Europe and Africa [8, 11, 12, 37]. On the other hand, the invasion to West Australia is a rather old event, having its roots in South Africa, while the different outbreaks in South Australia seem to have originated from Western Australia [9]. More extended sampling efforts from four continents, focusing on cytochrome oxidase I (COI) gene, clearly support the Afrotropical origin of the medfly that spread towards all other regions through recent, repeated invasions [36].

Our analysis included 15 medfly populations from Europe, Middle East, Australia, and the Americas (North, Central, and South). Sampling was expanded to regions previously not included in the population genetic studies of the species, covering Central America (with samples from Costa Rica, Honduras, and Nicaragua) and South America (with samples from Argentina and Bolivia) and more recent collections from previously studied regions. Our results are in accordance with the main proposed scenarios since a) samples from the Mediterranean basin cluster together, b) new samples from Central America are closely related to Mediterranean samples, c) the two samples from Australia are genetically very similar, which is consistent with a single invasion event (or of a single origin) and subsequent spread in different locations, and d) South America populations may form a discrete cluster, however not very different from the other medfly populations.

### Symbiotic diversity and structuring

Few studies have been performed in the medfly to characterize microbiota communities from natural populations, aiming mainly to support SIT applications. Most of them suggest that medfly symbiotic communities constitute mainly of Gammaproteobacteria, and more precisely from rather few Enterobacteriaceae genera. Among them, *Klebsiella oxytoca*, has been identified as a major symbiont of natural populations [38] and has been shown to affect different parameters of rearing and biological quality of released males when used as a supplement in larval and adult diet [19, 23]. Minor but persistent *Pseudomonas* communities have also been identified by different studies [17, 27, 28]. *Enterobacter* is also a key member of medfly gut microbiota community, especially in laboratory populations, and has also been shown to positively influence rearing and biological quality of males produced for release purposes when supplementing larval and adult diet [22–24]. Microbiota communities of reduced variability have been identified in long-established laboratory populations and strains of SIT importance (including the VIENNA 7 and VIENNA 8 GSS), a factor that could affect several aspects of the biology, physiology, nutrition and ecology of these strains [19, 21, 22, 26]. Different studies have also identified some other genera being usually present in natural populations of the species and in high relative abundances including *Acinetobacter* sp., *Providencia* sp., *Morganella* sp., and others [22, 27, 28]. However, knowledge on microbiota of medfly natural populations is rather limited since the majority has focused on laboratory strains of SIT importance [19, 21, 22, 26] or other domesticated populations [28]. Only few studies have focused on the characterization of microbiota from medfly wild populations. In this respect, few Enterobacteriaceae genera have been shown to constitute the majority of medfly gut microbiota collected in Israel More recently, Malacrino and his colleagues analysed the microbiota of medfly populations collected from one Italian region but from different hosts [27] and showed that a) gut microbiota communities of medfly natural populations in the area consisted mainly from Proteobacteria but a Firmicutes community with substantial relative abundance is present as well and b) both developmental stage and host are important for the structuring of gut microbiota.

Our analysis of the symbiotic profile of medfly expanded our knowledge on the microbiota structuring in natural populations of Europe, Australia, Middle East, and Central plus South America. Our data indicated that few Enterobacteriaceae genera (twelve) shaped symbiotic communities of the species, although many more are present in minor relative abundances in the different populations. This is in accordance with previous findings suggesting genera such as *Klebsiella, Providencia, Enterobacter, Morganella, Citrobacter,* and *Pantoea*, as being the major components of medfly microbiota communities [19, 22, 25, 26, 28, 38]. Furthermore, our analysis revealed the presence of *Rahnella* in the populations from Australia_1 (1%), Australia_2 (8.3%), Nicaragua (3.2%) and Spain (3%). This genus has been rarely identified in fruit flies, with one study showing its presence in *Anastrepha* species [39]. However, this is the first time that this bacterium has been identified in medfly. *Rahnella* has been found to be associated with *Dendroctonus pondorosae*, and it has been found to metabolize monoterpenes and diterpenes acids [40]. Persistent (but minor) *Pseudomonas* communities have also been described [17, 27] and this is the case for most of the samples in our study as well. However, the study of Malacrino and his colleagues [27] present a completely different microbiota profile of medflies from Italy since, besides Proteobacteria, an abundant Firmicutes community was present (~ 10%). Additionally, even within Proteobacteria, besides Gammaproteobacteria, there were abundant communities of Alpha-, Beta-, and Deltaproteobacteria.

Previous studies also indicate that geographic origin can explain the differential presence of bacteria genera among different studies [27]. However, our analysis of worldwide collected samples supports that most of the genera belong to Enterobacteriaceae and are common among different populations with the differential relative abundance being the factor leading the differentiation of the microbiota profiles. The sample from Honduras can be considered as an exception since Firmicutes prevailed but even in this case a) Firmicutes have also been described by previous studies [27, 28], and b) the remaining abundant genera, mainly Enterobacteriaceae, were present in the other populations as well and have also been described in previous studies [19, 21, 22, 26, 28, 38].

Albeit the Honduras population is genetically clustered with the rest Central American populations, their symbiotic profiles present major discrepancies. The genus *Exiguobacterium* is abundant in Honduras and absent or in extremely low abundance in the rest geographically neighbouring populations. Although not previously found in medfly, *Exiguobacterium* has been isolated from *Zeugodacus cucurbitae* during an effort to identify putative attractants but it has not been further utilized [41]. In our study, only nine individuals from Honduras were available and analysed. Unfortunately, the host is also unknown. Despite the abovementioned limitations, comparing the results at the genetic level and the variance at the symbiotic level between Honduras and the samples of Central American origin is noteworthy and calls for more extended sampling in this area to show whether this is a generalized symbiotic profile of medfly populations from Honduras.

### Factors shaping medfly microbiota communities

All pairwise comparisons among the different samples showed statistically significant differentiation of the symbiotic profiles. Therefore, geographic origin is an important factor and this has been shown for medfly and fruit flies in general [27, 28, 42]. Our analysis does not point to the sex as a factor contributing to the structuring and this is also well in agreement with previous studies in fruit flies [42, 43]. There are other factors known to influence microbiota structuring of fruit flies, such as the developmental stage, the age, and the host [22, 27, 42, 43]. Such factors have not been addressed in the present study, since only adults were used, either collected from traps or from fruits that emerged in the laboratory, which is the case for the Greek populations. Due to the lack of information on the collection strategy and the fact that in most cases adults collected from traps were used, the age of the collected adults and whether they had the chance to feed (or on what resources) after their emergence are not known. This fact limits our capacity to assess the diversity of the symbiotic communities at a lower level since we do not know which components and at which extent contributed to the formation of the symbiotic profiles we observed.

Recent studies also point to the possible impact of methodological approaches (such as samples’ preservation) on the results. It has been shown in medfly that sample preservation in 70% ethanol influences analysis of gut microbiota [28]. All the samples used in this study were stored either in 100% ethanol or propylene glycol for varying periods of time. Since samples were collected from different regions and for different purposes, it was not possible to collect and preserve samples using a universalized approach. Therefore, unified protocols for sample collection, preservation, and analysis are needed to allow unbiased comparisons between the different laboratories.

### Importance of genetic and microbiota profile for SIT applications

SIT, as well as other methodologies proposing population suppression through inducing sterility in natural populations using laboratory males, rely on the cost-efficient mass production and release of males that will be of high biological quality and competitiveness in the field. Cayol and colleagues studied wild and laboratory medfly populations and showed that mating compatibility was not negatively affected thus supporting the notion that the species has not yet evolved specific mating behaviours worldwide [44]. However, genetic differences between the mass-reared sterile males and the targeted natural population may lead to selective disadvantage during mating in the wild which can weaken SIT efficiency. Therefore, knowledge of the genetic structure of natural populations in the targeted areas is important. Continuous suppression without achieving eradication could, theoretically lead to the selection of genotypes in the natural population that are less prone to mating with mass-reared sterile males [3], therefore it is important to follow up population genetics in the targeted area, prior and after SIT application.

Performance in the field is an extremely important factor for the successful SIT application and can be affected both by inherent properties of the strains and the process of rearing. Rearing is a crucial step for SIT, and the initial fly material used in the rearing process as well as the genomic changes during the colonization process are important factors regarding the biological quality of the released individuals. Several studies have reported that life history traits can be affected during the laboratory adaptation process including reduction in developmental time, lifespan, dispersal ability and stress resistance, as well as early fertility and increased fecundity [32, 45]. Any decrease in the quality of desirable biological traits may put in risk the efficiency of the SIT operational programs. The loss of the genetic diversity might be a rapid process for some species taking place quite early after the introduction of the wild population in the laboratory [31, 45]. Therefore, it is essential to develop a strategy that will allow maintaining genetic diversity by enriching the mass-reared colonies with fresh fly material introduced from the wild [46]. Medfly has been a model for how larval or adult diets supplemented with probiotics can boost different parameters, such as productivity, mating competitiveness, longevity, and flight ability, with Enterobacteriaceae species belonging to *Pantoea, Enterobacter,* and *Klebsiella* genera showing encouraging results [16, 19–24]. Recent studies in other fruit flies also point to the importance of microbiota for the biological quality of released males [47, 48]. However similar bacteria may not have similar effects. Therefore, identifying microbiota of a species across its range (geographical or plant host) can provide evidence either for the core microbiome that is needed for medfly or for the importance of specific bacterial strains under certain conditions.

## Conclusions

SIT depends on the cost-efficient production and high performance of laboratory-reared males in the field. Genetic structure and symbiotic profile can influence mating compatibility of laboratory and natural populations thus affecting SIT performance. The present study expanded our knowledge on the genetic structure of medfly natural populations by including under-sampled regions and revisiting previously analyzed populations. Our findings are in accordance with the prevailing scenarios for the invasion events and pathways of this cosmopolitan species and did not find any indication for the presence of extremely sub-structured populations in the dataset analyzed. The symbiotic analysis shed light on the structure of the medfly natural populations’ microbiota, since eleven new samples were analyzed collected from 9 different countries. Our analysis pointed to the presence of a symbiotic community of reduced diversity, since 12 genera (mainly Enterobacteriaceae) are the key components of all symbiotic communities. However, the presence of a well differentiated microbiota community in medflies from Honduras calls for additional analysis, since important questions regarding whether it is a universal profile in the area or how that might affect insect’s behavior remain open. The genetic and the symbiotic profile of pest species in areas targeted for SIT must be studied and compared with the profiles of laboratory strains reared for release purposes. That way, either probiotic supplements or enrichment from the wild protocols can be applied to boost the efficiency of SIT.

## Methods

The genetic structure of fifteen medfly populations was analyzed with a set of eight microsatellite markers. Symbiotic microbiota communities were analyzed for eleven of them with high throughput Illumina sequencing of the 16S rRNA gene.

### Samples collection, DNA extraction, and storage

Adult females and males were sampled and stored in 96–100% ethanol or propylene glycol at − 20 °C until DNA extraction. Samples’ details are presented in Additional file 1 Table S1. Total DNA was extracted from individual adults using the “Extract Me Genomic DNA kit” (DNA Gdansk, Poland) according to the manufacturer’s instructions. Prior to DNA extraction, samples were surface sterilized through dipping in 70% ethanol. All fifteen populations were included in the genetic analysis, while the symbiotic communities were studied in eleven populations. Regarding the eleven population being in common in both studies, the same DNA samples were used. Microbiota structuring was analyzed for fewer populations for logistic reasons, trying to keep the worldwide distribution of the samples.

### Microsatellite analysis

#### PCR reactions, allele scoring, and genotyping

The following microsatellite markers were used in the present analysis: *Medflymic30*, *Medflymic43*, *Medflymic74*, *Medflymic78* [49], *Ccmic6*, *Ccmic9*, *Ccmic14*, and *Ccmic32* [7, 50]. All forward primers of the 8 microsatellite loci were fluorescently labelled (Thermo Fisher Scientific, Waltham, Massachusetts, USA) as shown in Additional file 2 Table S2. PCR reactions were performed in a total volume of 20 μl, containing 1x reaction buffer (Solis BioDyne OÜ, Tartu, Estonia), 0.2 mM of each dNTP (Thermo Fisher Scientific, Waltham, Massachusetts, USA), 0.5 μM of each primer, 1 μL of DNA template, and 1u of HOT FIREPol® DNA Polymerase (Solis BioDyne OÜ, Tartu, Estonia). The PCR cycling conditions included an initial step of denaturing at 95 °C for 5 min, followed by 35 cycles of 95 °C for 50 s, 57 °C or 60 °C for 50 s, 72 °C for 1 min, and a final elongation step at 72 °C for 10 min. Prior to the fragment analysis, each fluorescently labelled PCR product was combined in a mix including four different labelled products (see Additional file 2 Table S2 for the two sets of mixed PCR products). Genescan 500 LIZ (500 bp) (Applied Biosystems) was used as an internal standard size marker. Fragment analysis was performed by Macrogen (Macrogen, Seoul, Korea). GENEIOUS version R10 (https://www.geneious.com) loaded with the Geneious microsatellite plugin (https://www.geneious.com/plugins/microsatellite-plugin/) was used to visualize the traces, fit the Genescan 500 LIZ ladder, call peaks, predict bins, and create the amplicon size scoring matrix.

#### Genetic variability and data analysis

Genetic variability parameters, including the effective number of alleles (Ne), the observed (Ho), and expected (He) heterozygosities under Hardy- Weinberg equilibrium, were measured using GenAlEx version 6.51 [51, 52]. Analysis of Molecular Variance (AMOVA) was performed in Genalex 6.51 to estimate the percentage of variance attributed to within and between population variability. The pairwise population genetic distance analysis was calculated according to Nei [53] using GenAlEx 6.51. Genetic clusters were determined using the principal coordinates analysis (PCA) by GenAlEx 6.51. The pairwise population matrix of Nei genetic distance was used as an input for PCA. The STRUCTURE software version 2.3.4 [33, 54–56] was used to perform a Bayesian clustering analysis. STRUCTURE software detects allele frequency differences and assigns individuals to population clusters. The no-admixture model with an assumption of correlated allele frequency among populations [54] was used, with a burn-in period of 100, 000 and 100, 000 Markov Chain Monte Carlo (MCMC) repetitions after the initial burn-in. The no-admixture model was chosen based on the notion that the samples were collected in distinct locations. We assumed *K* = 1 to 10 and performed 20 repetitions for each potential *K*. STRUCTURE HARVESTER [57] was used to estimate the modified *K*-value by calculating ln(*K*) and delta *K*, based on the modification described by Evanno et al. [34]).

### 16S rRNA gene analysis

DNAs were tested for quality and quantity using a Q5000 micro-volume UV-Vis spectrophotometer (Quawell Technology, San Jose, CA, USA) and were stored in Eppendorf tubes at − 20 °C until PCR amplification and amplicon sequencing analysis were carried out.

### Library preparation, Illumina MiSeq sequencing, and bioinformatics analysis

For the amplicon sequencing analysis, the hypervariable V3-V4 region of the bacterial 16S rRNA gene was amplified using MiSeq universal primers 341F and 805R [58]. The first PCR reaction was performed in 25 μL reaction mixtures containing 2.5 μL KAPA Taq buffer 10x, 0.25 μL dNTPs (25 mM), 0.25 μL of KAPA Taq, 0.5 μL of the forward primer (25 μM), 0.5 μL of the reverse primer (25 μM), 1 μL of template DNA solution and was finalized with 20 μL sterile deionized water. The PCR temperature profile was 95 °C for 5 min followed by 35 cycles of 95 °C for 30 s, 30 s at 55 °C, 1 min at 72 °C and a final extension step of 72 °C for 5 min. PCR products were electrophoresed on a 1.5% agarose gel in order to examine the presence and size of the amplified fragments. Negative controls were included in DNA extractions and PCRs were performed under the same conditions as the rest of the samples but without any genetic material. No amplicons were obtained from these negative controls. To include the indexes as well as the Illumina adaptors, a second PCR was performed in 50 μL volume containing 5 μL KAPA Taq buffer 10x, 0.4 μL dNTPs (25 mM), 0.2 μL of KAPA Taq, 5 μL of the forward index primer (10 μM), 5 μL of the reverse index primer (10 μM), 2 μL of the cleaned PCR product diluted up to 10 ng.μL^− 1^ and 32.4 μL sterile deionized water. The temperature profile used for the PCR was: 95 °C for 3 min followed by 8 cycles of 95 °C for 30 s, 30 s at 55 °C, 1 min at 72 °C and a final extension step of 72 °C for 3 min. The resulting amplicons were purified using the NucleoMag NGS Clean-up and Size Selection kit (Macherey-Nagel, Düren, Germany) following the manufacturer’s instructions. Indexed amplicons from all samples examined were mixed in equimolar ratio (8 nM) and sequencing was performed by Macrogen using a 2x300bp pair-end kit on a MiSeq platform.

After sequencing, bioinformatic analysis was performed using USEARCH version 11 [59] and Quantitative Insights Into Microbial Ecology (QIIME2) distribution 2019.1 [60]. Briefly, paired-end reads were assembled, trimmed by length using the usearch -fastq_mergepairs option, then, the quality of assembled sequences was improved using –fastq_filter, followed by finding unique read sequences and abundances using –fastx_uniques option. Sequences were clustered into operational taxonomic units (OTUs) with -cluster_otus command based on 97% OTU clustering using UPARSE algorithm [61]. Cross-talk errors were identified and filtered with –uncross option based on UNCROSS2 algorithm [62]. Taxonomy was assigned with Qiime2 based on BLAST+ algorithm [63] against SILVA 128 release database [64]. For those OTUs that taxonomy could not be assigned at genus level improved taxonomic assignment were performed using SINA and ARB [65, 66].

Richness, Simpson, Shannon, and Evenness indices of alpha diversity, which reflect the diversity of individual samples were calculated based on the “diversity” function of the “vegan” R package and plotted using “ggplot” function of the “ggplot2” package. Pair-wise ANOVA was used to identify significant differences of alpha diversity indices between the different locations. Beta diversity was analyzed to evaluate the similarity of bacterial communities from different locations using Generalized UniFrac distance [67] and visualized via metric and non-metric multidimensional scaling plots. A permutational multivariate analysis of variance using distance matrices was calculated using “adonis” function from “vegan” R package to determine significance differences between the separated groups. Multidimensional scaling (MDS) analysis and the multidimensional plots as implemented in PRIMER version 6+ [68] were used as well. Permutational Multivariate Analysis of Variance (PERMANOVA) analyses were applied to Bray-Curtis similarity matrices to compute similarities between groups. Differences in community structure were viewed using the constrained ordination technique CAP (Canonical Analysis of Principal Coordinates), using the CAP classification success rate and CAP trace_Q_m’HQ_m_ statistics, and were performed with 9999 permutations within PRIMER version 6+ [69]. Cap analysis was performed using the Bray-Curtis similarity matrices.

## Supplementary Information

**Additional file 1:** **Table S1.** Samples analyzed.

**Additional file 2:** **Table S2.** Microsatellite markers used and diversity indices.

**Additional file 3:** **Table S3.** Deviations from HWE.

**Additional file 4:** **Figure S1.** Analysis of Molecular Variance.

**Additional file 5:** **Table S4.** Pairwise genetic distances matrix (Nei 1972).

**Additional file 6:** **Figure S2.** Identification of ‘true’ number of populations using the modification of Evanno and colleagues (2005).

**Additional file 7:** **Figure S3.** Relative abundances of medfly microbiota at A) Phylum level, B) Class level, and C) Genus level.

**Additional file 8:** **Figure S4.** Alpha diversity of the different samples and pairwise comparisons A) Richness index, B) Shannon index.

**Additional file 9:** **Table S5.** PERMANOVA pairwise values (corrected *p*-values).

**Additional file 10:** **Figure S5.** MDS plot of the microbial profiles of male and female medflies.

**Additional file 11:** **Figure S6.**The different OTUs (putative species) assigned to *Klebsiella* genus and their relative abundance in the medfly natural populations.

**Additional file 12:** **Figure S7.**The different OTUs (putative species) assigned to *Morganella* genus and their relative abundance in the medfly natural populations.

**Additional file 13:** **Figure S8.**The different OTUs (putative species) assigned to *Providencia* genus and their relative abundance in the medfly natural populations.

**Additional file 14:** **Figure S9.**The different OTUs (putative species) assigned to *Enterobacter* genus and their relative abundance in the medfly natural populations.

## Data Availability

All data generated during this study are included in this published article and its supplementary information files. The 16S rRNA gene sequence datasets generated for this study can be found in NCBI, PRJNA662444.

## Abbreviations

SIT: Sterile Insect Technique
AW-IPM: Area-Wide Integrated Pest Management
GSS: Genetic Sexing Strain(s)
HWE: Hardy-Weinberg Equilibrium
AMOVA: Analysis of Molecular Variance
ANOVA: Analysis of Variance
PCoA: Principal Coordinates Analysis
CAP: Canonical Analysis of Principal Coordinates
MDS: Multi-Dimensional Scaling
NMDS: Non-metric Multidimensional Scaling
