# Supplementary material for: Genetic structure and symbiotic profile of worldwide natural populations of the Mediterranean fruit fly, Ceratitis capitata
Source: BMC Genet. 2020 Dec 18;21(Suppl 2):128. doi: 10.1186/s12863-020-00946-z (PMC7747371; doi:10.1186/s12863-020-00946-z)
Supplement: Supplementary file 1 — Additional file 1: Table S1. Samples analyzed. [file 12863_2020_946_MOESM1_ESM.docx]

Additional File 1 Table S1: Samples analyzed

| No |  | Population | | | | Genetic analysis (N) | | Symbiotic analysis (N) | |
| --- | --- | --- | --- | --- | --- | --- | --- | --- | --- |
|  |  | **code** | Origin | Host | Year | M | F | M | F |
| 1 | Europe | Greece1 | Greece, Volos | Bitter Oranges | 2013 | 15 | 15 | 14 | 9 |
| 2 |  | Greece2 | Greece, Volos | Figs | 2013 | 15 | 9 | 14 | 9 |
| 3 |  | Spain | Spain, Valencia | Unknown | 2012 | 34 | 16 | 11 | 14 |
| 4 |  | Croatia | Croatia | Unknown | 2012 | 14 | 15 | 14 | 10 |
|  |  |  |  |  |  |  |  |  |  |
| 5 | Asia/Middle East | Israel | Israel | Pear | 2012 | 20 | 20 | 20 | 20 |
|  |  |  |  |  |  |  |  |  |  |
| 6 | Australia | Australia1 | Australia, Broome | Kumquat | 2009 | 15 | 5 | 15 | 5 |
| 7 |  | Australia2 | Australia, Gosnell | Loquat | 2012 | 14 | 15 | 14 | 15 |
|  |  |  |  |  |  |  |  |  |  |
| 8 | North America | Hawaii | USA, Hawaii | Unknown | 2013 | 10 | 14 | nt | nt |
|  |  |  |  |  |  |  |  |  |  |
| 9 | Central America | El Salvador | El Salvador | Unknown | 2013 | 11 | 13 | nt | nt |
| 10 |  | Honduras | Honduras | Unknown | 2012 | 4 | 5 | 4 | 5 |
| 11 |  | Nicaragua | Nicaragua, IPA | Unknown | 2012 | 29 | 0 | 29 | 0 |
| 12 |  | Costa Rica | Costa Rica | Unknown | 2013 | 12 | 12 | nt | nt |
|  |  |  |  |  |  |  |  |  |  |
| 13 | South America | Argentina | Argentina, San Juan | Unknown | 2016 | 20 | 4 | 12 | 3 |
| 14 |  | Brazil | Brazil, Pernambuko | Guava | 2013 | 14 | 14 | 14 | 14 |
| 15 |  | Bolivia | Bolivia | Unknown | 2011 | 4 | 20 | nt | nt |
|  | | | | | | | | | |
