# Supplementary material for: Genetic structure and symbiotic profile of worldwide natural populations of the Mediterranean fruit fly, Ceratitis capitata
Source: BMC Genet. 2020 Dec 18;21(Suppl 2):128. doi: 10.1186/s12863-020-00946-z (PMC7747371; doi:10.1186/s12863-020-00946-z)
Supplement: Supplementary file 2 — Additional file 2: Table S2. Microsatellite markers used and diversity indices. [file 12863_2020_946_MOESM2_ESM.docx]

Additional File 2 Table S2: Microsatellite markers used and diversity indices

| **Locus** | **Forward & reverse**  **primer sequences 5’-3’** | **L** | **T** | **Set** | N | Na | Ne | Ho | He | HWE |
| --- | --- | --- | --- | --- | --- | --- | --- | --- | --- | --- |
| *Medflymic43* | TTTTCGAACGGCTGCATC  TTAGAGGCAAGCCACCAGG | 6-FAM | 60 | 1 | 22.267 | 3.867 | 2.410 | 0.460 | 0.516 | 6/15 |
| *Ccmic6* | AAGGTAGCCAGCAGTGTCTACG  ACGAATGGGAGTTATTCATACTGC | VIC | 57 |  | 24.933 | 2.800 | 1.535 | 0.282 | 0.280 | 1/15 |
| *Medflymic30* | TACTGGACAACGGGTTAACAGC  TTTTATGTTCAACGCTGCGAC | NED | 57 |  | 24.067 | 2.000 | 1.666 | 0.412 | 0.378 | 1/15 |
| *Ccmic32* | ACCACCCAATAACTTCATA  GCTTTCATCATCCGTTCC | PET | 57 |  | 26.333 | 4.133 | 2.627 | 0.675 | 0.584 | 9/15 |
| *Ccmic9* | GAAGTGACTCATATTTTTAGGAACGA  TCTTTCTTTCATACTCACTCATTTC | 6-FAM | 57 | 2 | 25.200 | 3.267 | 1.674 | 0.295 | 0.324 | 5/15 |
| *Medflymic78* | ATTTGCCCGTCATTCAAACAAC  ATTTATACACCCAGTCATGCCC | VIC | 57 |  | 22.267 | 2.533 | 1.816 | 0.604 | 0.442 | 7/15 |
| *Ccmic14* | AATTCAGATACACGCTCACAAG  TCGTATTGCTATGCGCATAT | NED | 57 |  | 20.067 | 1.533 | 1.048 | 0.151 | 0.167 | 1/15 |
| *Ccmic14* | TCAAAGAAACAAAGAGGCGTG  TAAGCAGCAGACACAAGTGTTC | PET | 57 |  | 24.133 | 2.667 | 1.843 | 0.278 | 0.357 | 3/15 |
| *average* | | | | | *23.658* | *2.850* | *1.827* | *0.395* | *0.381* |  |
