# Supplementary material for: Genetic structure and symbiotic profile of worldwide natural populations of the Mediterranean fruit fly, Ceratitis capitata
Source: BMC Genet. 2020 Dec 18;21(Suppl 2):128. doi: 10.1186/s12863-020-00946-z (PMC7747371; doi:10.1186/s12863-020-00946-z)
Supplement: Supplementary file 3 — Additional file 3: Table S3. Deviations from HWE. [file 12863_2020_946_MOESM3_ESM.docx]

Additional File 3 Table S3: Deviations from HWE

|  | *Medflymic43* | *Ccmic6* | *Medflymic30* | *Ccmic32* | *Ccmic9* | *Medflymic78* | *Ccmic14* | *Medflymic74* |
| --- | --- | --- | --- | --- | --- | --- | --- | --- |
| Greece1 | ns | ns | ns | ** | ns | *** | ns | ns |
| Greece2 | * | ns | ns | * | * | *** | ns | ns |
| Spain | *** | ns | ns | ns | ns | *** | ns | ns |
| Croatia | *** | ns | ns | * | *** | * | ns | ns |
| Israel | *** | ns | ns | ns | ** | ns | ns | ns |
| Australia1 | ** | ns | * | * | *** | ns | nt | nt |
| Australia2 | ns | ns | ns | *** | *** | *** | ns | nt |
| Hawaii | ns | ns | ns | ns | ns | *** | ns | ns |
| El Salvador | ns | nt | ns | *** | ns | ns | ns | *** |
| Honduras | ns | nt | nt | ns | nt | nt | ns | ns |
| Nicaragua | ns | ns | ns | * | ns | ns | ns | ns |
| Costa Rica | ns | nt | ns | ns | ns | ns | ns | ns |
| Argentina | ns | ns | ns | ns | ns | *** | ns | nt |
| Brazil | *** | ns | ns | ** | ns | ns | ** | ** |
| Bolivia | nt | * | ns | ** | ns | ns | ns | *** |

**ns=not significant, * P<0.05, ** P<0.01, *** P<0.001, nt=not tested (monomorphic)**
