# Supplementary figures and images for: Genetic structure and symbiotic profile of worldwide natural populations of the Mediterranean fruit fly, Ceratitis capitata
Source: BMC Genet. 2020 Dec 18;21(Suppl 2):128. doi: 10.1186/s12863-020-00946-z (PMC7747371; doi:10.1186/s12863-020-00946-z)

Additional File 4 Figure S1: Analysis of Molecular Variance


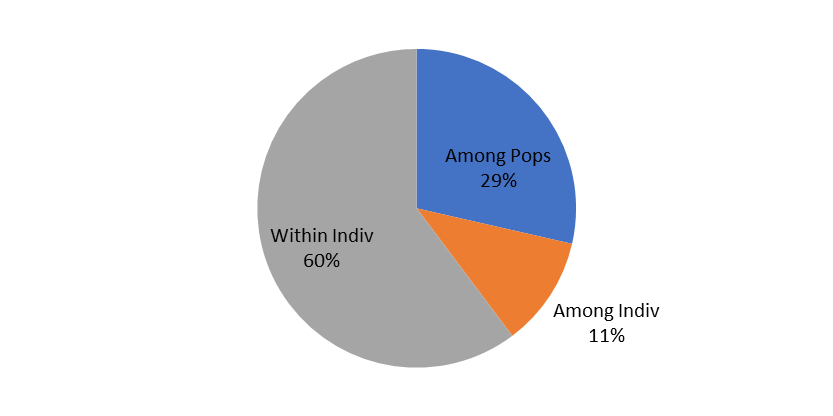

Supplement: Supplementary file 4 — Additional file 4: Figure S1. Analysis of Molecular Variance. [file 12863_2020_946_MOESM4_ESM.docx]

Additional File 10 Figure S5: MDS plot of the microbial profiles of male and female medflies


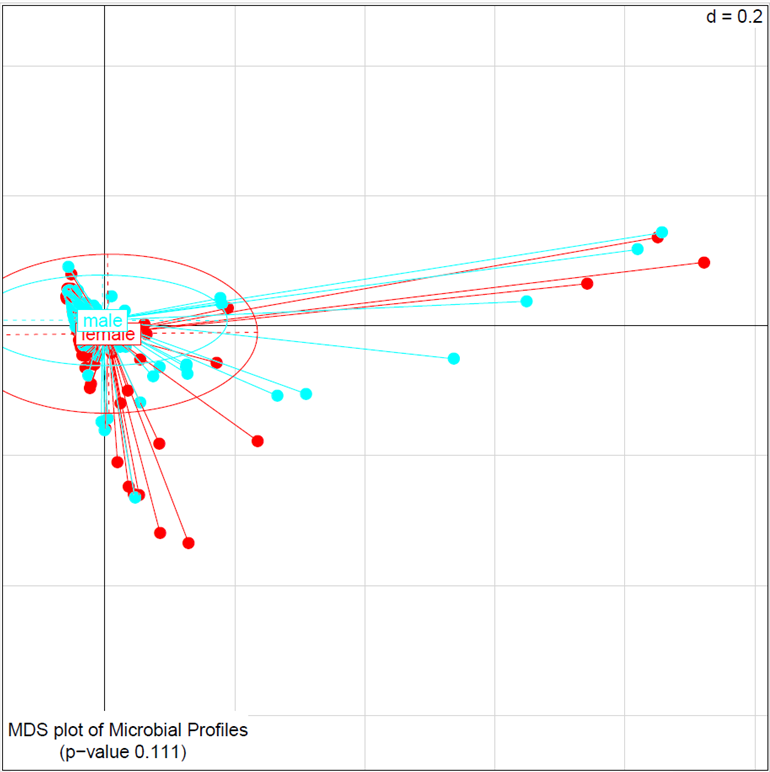

Supplement: Supplementary file 10 — Additional file 10: Figure S5. MDS plot of the microbial profiles of male and female medflies. [file 12863_2020_946_MOESM10_ESM.docx]
