# Supplementary material for: Genetic structure and symbiotic profile of worldwide natural populations of the Mediterranean fruit fly, Ceratitis capitata
Source: BMC Genet. 2020 Dec 18;21(Suppl 2):128. doi: 10.1186/s12863-020-00946-z (PMC7747371; doi:10.1186/s12863-020-00946-z)
Supplement: Supplementary file 5 — Additional file 5: Table S4. Pairwise genetic distances matrix (Nei 1972). [file 12863_2020_946_MOESM5_ESM.docx]

Additional File 5 Table S4: Pairwise genetic distances matrix (Nei 1972)

|  | 1 | 2 | 3 | 4 | 5 | 6 | 7 | 8 | 9 | 10 | 11 | 12 | 13 | 14 | 15 |
| --- | --- | --- | --- | --- | --- | --- | --- | --- | --- | --- | --- | --- | --- | --- | --- |
| Greece1 (1) |  | 0.133 | 0.074 | 0.036 | 0.119 | 0.189 | 0.156 | 0.270 | 0.171 | 0.710 | 0.096 | 0.168 | 0.386 | 0.399 | 0.281 |
| Greece2 (2) |  |  | 0.139 | 0.118 | 0.192 | 0.298 | 0.254 | 0.213 | 0.184 | 0.803 | 0.174 | 0.212 | 0.216 | 0.626 | 0.386 |
| Spain (3) |  |  |  | 0.035 | 0.097 | 0.183 | 0.177 | 0.298 | 0.087 | 0.445 | 0.070 | 0.084 | 0.302 | 0.382 | 0.202 |
| Croatia (4) |  |  |  |  | 0.069 | 0.135 | 0.102 | 0.265 | 0.082 | 0.588 | 0.077 | 0.102 | 0.340 | 0.438 | 0.268 |
| Israel (5) |  |  |  |  |  | 0.185 | 0.161 | 0.360 | 0.206 | 0.591 | 0.144 | 0.069 | 0.412 | 0.506 | 0.192 |
| Australia1 (6) |  |  |  |  |  |  | 0.032 | 0.330 | 0.349 | 0.763 | 0.259 | 0.222 | 0.521 | 0.459 | 0.451 |
| Australia2 (7) |  |  |  |  |  |  |  | 0.336 | 0.316 | 0.788 | 0.215 | 0.198 | 0.436 | 0.523 | 0.383 |
| Hawaii (8) |  |  |  |  |  |  |  |  | 0.366 | 0.963 | 0.324 | 0.385 | 0.293 | 0.397 | 0.605 |
| El Salvador (9) |  |  |  |  |  |  |  |  |  | 0.347 | 0.057 | 0.082 | 0.320 | 0.467 | 0.315 |
| Honduras (10) |  |  |  |  |  |  |  |  |  |  | 0.302 | 0.389 | 0.902 | 1.105 | 0.910 |
| Nicaragua (11) |  |  |  |  |  |  |  |  |  |  |  | 0.052 | 0.348 | 0.473 | 0.330 |
| Costa Rica (12) |  |  |  |  |  |  |  |  |  |  |  |  | 0.348 | 0.440 | 0.309 |
| Argentina (13) |  |  |  |  |  |  |  |  |  |  |  |  |  | 0.427 | 0.207 |
| Brazil (14) |  |  |  |  |  |  |  |  |  |  |  |  |  |  | 0.341 |
| Bolivia (15) |  |  |  |  |  |  |  |  |  |  |  |  |  |  |  |
