# Supplementary material for: Genetic structure and symbiotic profile of worldwide natural populations of the Mediterranean fruit fly, Ceratitis capitata
Source: BMC Genet. 2020 Dec 18;21(Suppl 2):128. doi: 10.1186/s12863-020-00946-z (PMC7747371; doi:10.1186/s12863-020-00946-z)

Additional File 6 Figure S2: Identification of ‘true’ number of populations using the modification of Evanno and colleagues (2005)


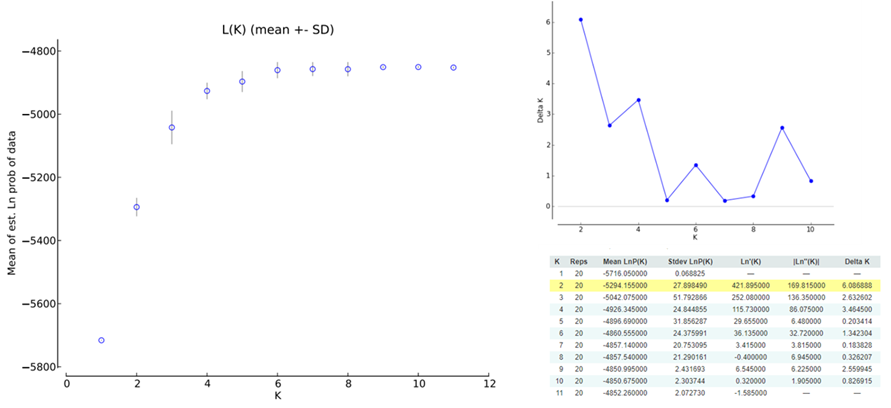

Supplement: Supplementary file 6 — Additional file 6: Figure S2. Identification of ‘true’ number of populations using the modification of Evanno and colleagues (2005). [file 12863_2020_946_MOESM6_ESM.docx]
