# Supplementary material for: Genetic structure and symbiotic profile of worldwide natural populations of the Mediterranean fruit fly, Ceratitis capitata
Source: BMC Genet. 2020 Dec 18;21(Suppl 2):128. doi: 10.1186/s12863-020-00946-z (PMC7747371; doi:10.1186/s12863-020-00946-z)

Additional File 7 Figure S3: Relative abundances of medfly microbiota at A) Phylum level, B) Class level, and C) Genus level


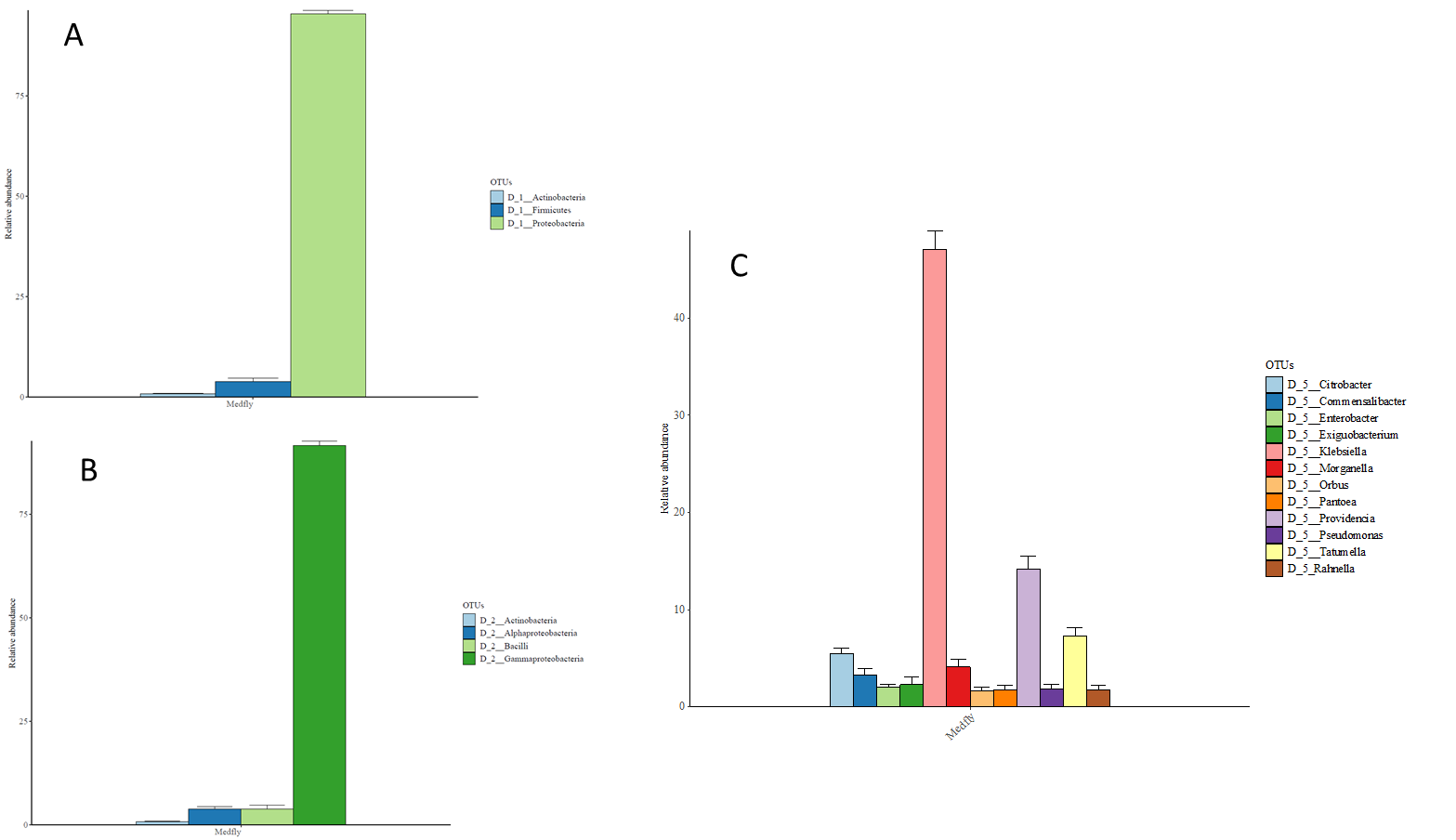

Supplement: Supplementary file 7 — Additional file 7: Figure S3. Relative abundances of medfly microbiota at A) Phylum level, B) Class level, and C) Genus level. [file 12863_2020_946_MOESM7_ESM.docx]
