# Supplementary material for: Genetic structure and symbiotic profile of worldwide natural populations of the Mediterranean fruit fly, Ceratitis capitata
Source: BMC Genet. 2020 Dec 18;21(Suppl 2):128. doi: 10.1186/s12863-020-00946-z (PMC7747371; doi:10.1186/s12863-020-00946-z)

Additional File 8 Figure S4: Alpha diversity of the different samples and pairwise comparisons A) Richness index, B) Shannon index


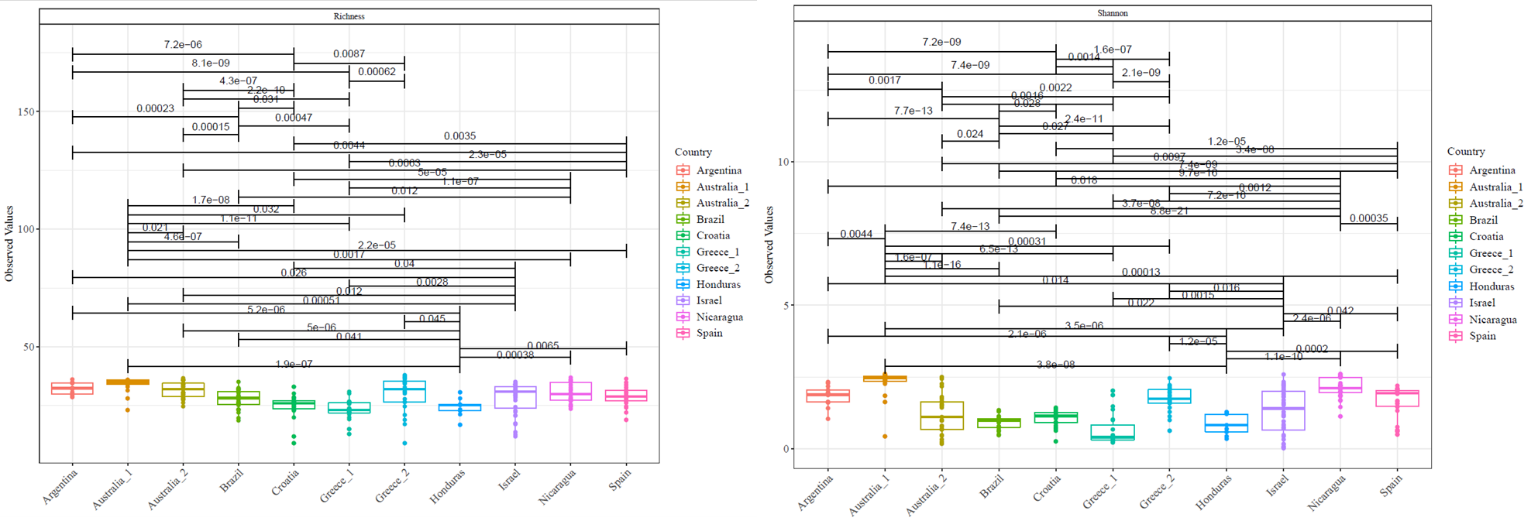

Supplement: Supplementary file 8 — Additional file 8: Figure S4. Alpha diversity of the different samples and pairwise comparisons A) Richness index, B) Shannon index. [file 12863_2020_946_MOESM8_ESM.docx]
