# Supplementary material for: Genetic structure and symbiotic profile of worldwide natural populations of the Mediterranean fruit fly, Ceratitis capitata
Source: BMC Genet. 2020 Dec 18;21(Suppl 2):128. doi: 10.1186/s12863-020-00946-z (PMC7747371; doi:10.1186/s12863-020-00946-z)
Supplement: Supplementary file 9 — Additional file 9: Table S5. PERMANOVA pairwise values (corrected p-values). [file 12863_2020_946_MOESM9_ESM.docx]

Additional File 9 Table S5: PERMANOVA pairwise values (corrected p-values)

|  | Greece1 | Greece2 | Spain | Croatia | Israel | Australia1 | Australia2 | Honduras | Nicaragua | Argentina | Brazil |
| --- | --- | --- | --- | --- | --- | --- | --- | --- | --- | --- | --- |
| Greece1 |  | 0.0013 | 0.0077 | 0.0012 | 0.0012 | 0.0013 | 0.0012 | 0.0012 | 0.0012 | 0.0012 | 0.0012 |
| Greece2 |  |  | 0.0023 | 0.0012 | 0.0013 | 0.0013 | 0.0012 | 0.0013 | 0.0012 | 0.0042 | 0.0012 |
| Spain |  |  |  | 0.0022 | 0.0023 | 0.0034 | 0.0012 | 0.0012 | 0.015 | 0.0076 | 0.0012 |
| Croatia |  |  |  |  | 0.0011 | 0.0013 | 0.0012 | 0.0012 | 0.0012 | 0.0012 | 0.0012 |
| Israel |  |  |  |  |  | 0.0012 | 0.0012 | 0.0012 | 0.0012 | 0.0013 | 0.0012 |
| Australia1 |  |  |  |  |  |  | 0.0013 | 0.0013 | 0.0024 | 0.0013 | 0.0012 |
| Australia2 |  |  |  |  |  |  |  | 0.0012 | 0.0012 | 0.0012 | 0.0013 |
| Honduras |  |  |  |  |  |  |  |  | 0.0012 | 0.0012 | 0.0013 |
| Nicaragua |  |  |  |  |  |  |  |  |  | 0.0012 | 0.0012 |
| Argentina |  |  |  |  |  |  |  |  |  |  | 0.0012 |
| Brazil |  |  |  |  |  |  |  |  |  |  |  |
