# Supplementary material for: Genetic structure and symbiotic profile of worldwide natural populations of the Mediterranean fruit fly, Ceratitis capitata
Source: BMC Genet. 2020 Dec 18;21(Suppl 2):128. doi: 10.1186/s12863-020-00946-z (PMC7747371; doi:10.1186/s12863-020-00946-z)

Additional File 11 Figure S6: The different OTUs (putative species) assigned to *Klebsiella* genus and their relative abundance in the medfly natural populations


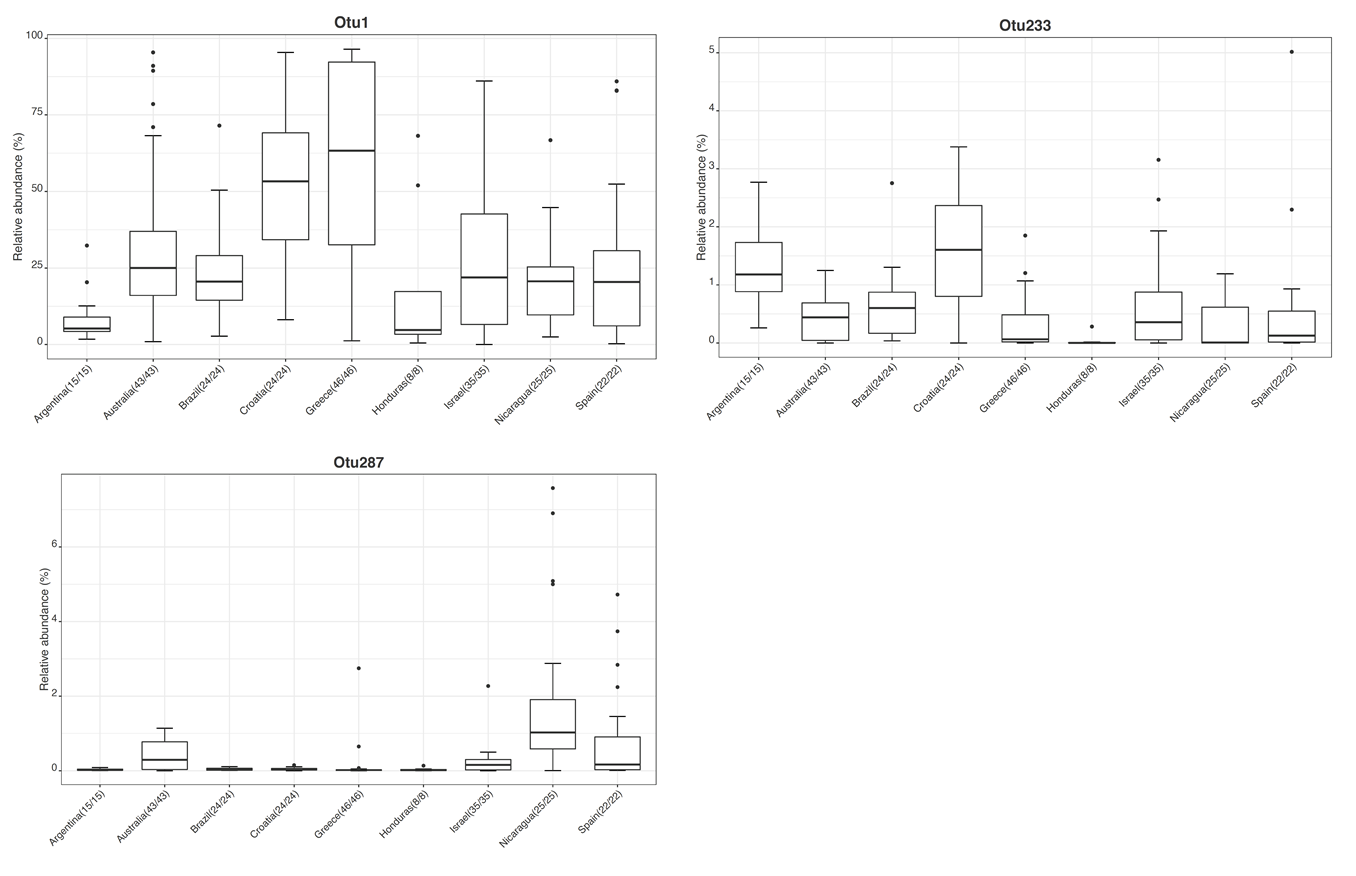

Supplement: Supplementary file 11 — Additional file 11: Figure S6.The different OTUs (putative species) assigned to Klebsiella genus and their relative abundance in the medfly natural populations. [file 12863_2020_946_MOESM11_ESM.docx]
