# Supplementary material for: Genetic structure and symbiotic profile of worldwide natural populations of the Mediterranean fruit fly, Ceratitis capitata
Source: BMC Genet. 2020 Dec 18;21(Suppl 2):128. doi: 10.1186/s12863-020-00946-z (PMC7747371; doi:10.1186/s12863-020-00946-z)

Additional File 12 Figure S7: The different OTUs (putative species) assigned to *Morganella* genus and their relative abundance in the medfly natural populations


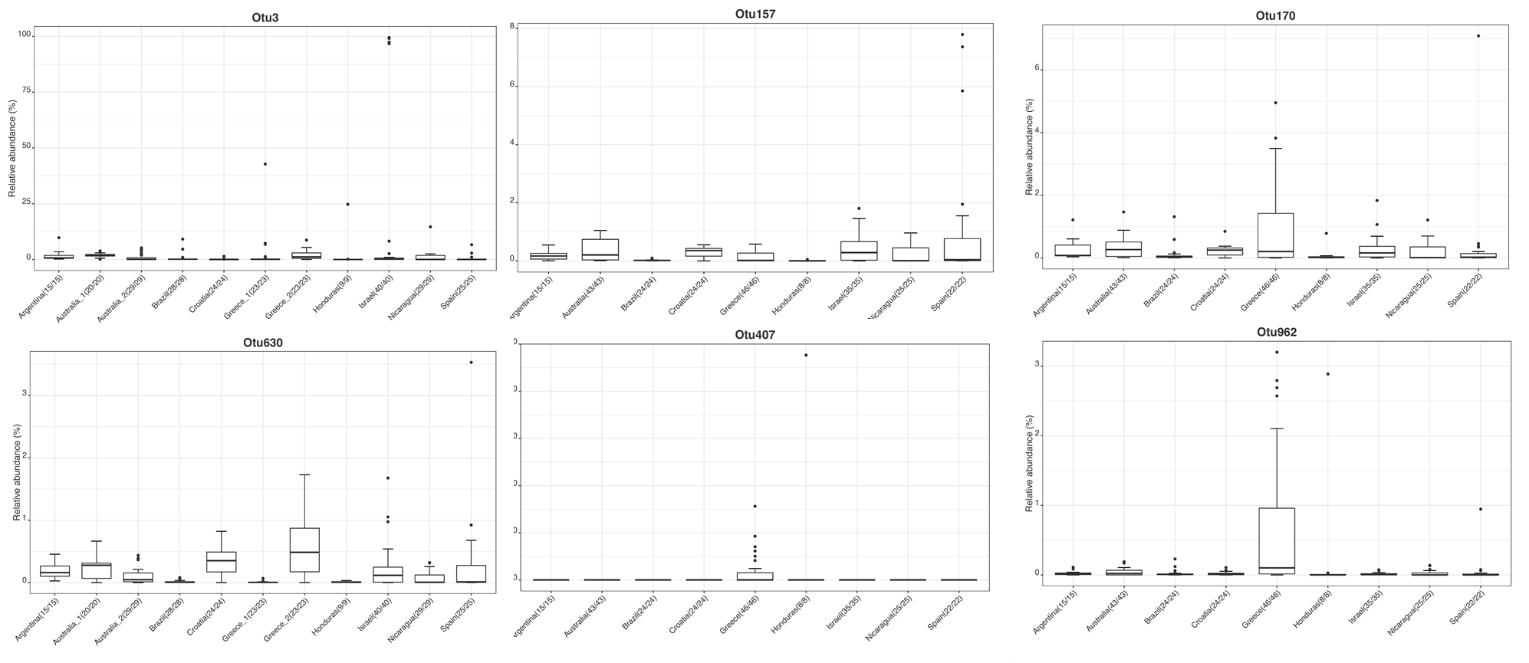

Supplement: Supplementary file 12 — Additional file 12: Figure S7.The different OTUs (putative species) assigned to Morganella genus and their relative abundance in the medfly natural populations. [file 12863_2020_946_MOESM12_ESM.docx]
