# Supplementary material for: Genetic structure and symbiotic profile of worldwide natural populations of the Mediterranean fruit fly, Ceratitis capitata
Source: BMC Genet. 2020 Dec 18;21(Suppl 2):128. doi: 10.1186/s12863-020-00946-z (PMC7747371; doi:10.1186/s12863-020-00946-z)

Additional File 13 Figure S8: The different OTUs (putative species) assigned to *Providencia* genus and their relative abundance in the medfly natural populations


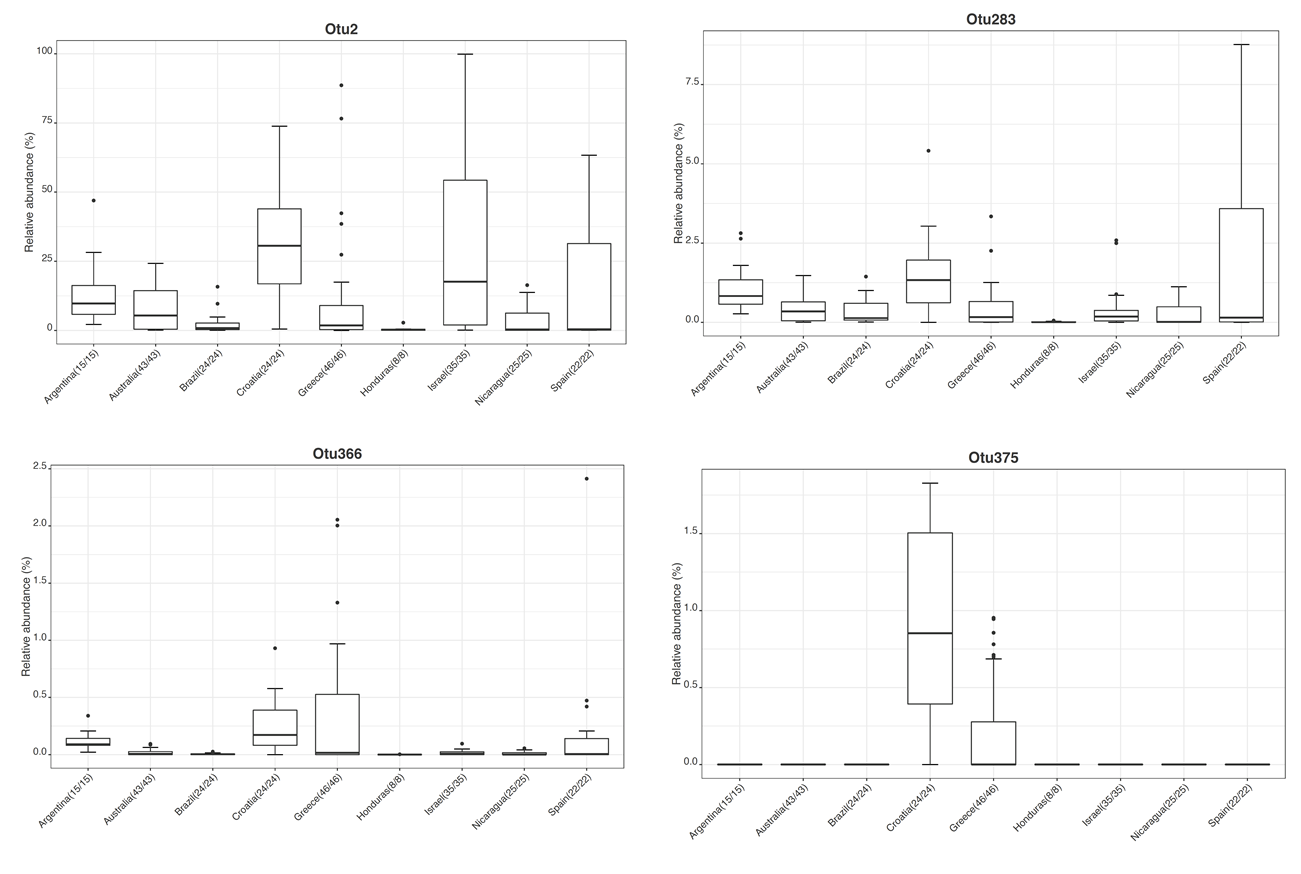

Supplement: Supplementary file 13 — Additional file 13: Figure S8.The different OTUs (putative species) assigned to Providencia genus and their relative abundance in the medfly natural populations. [file 12863_2020_946_MOESM13_ESM.docx]
